# Short Sleep Duration as a Risk Factor of Cardiovascular Disease in Korean Adults: Secondary Analysis of the Fifth Korean National Health and Nutrition Examination Survey

**Published:** 2019-07

**Authors:** Wonjung NOH, Heakyung MOON

**Affiliations:** 1. College of Nursing, Gachon University, Incheon, Republic of Korea; 2. College of Nursing, Hoseo University, Chungnam, Republic of Korea

**Keywords:** Cardiovascular disease, Hypertension, Korea, Sleep

## Abstract

**Background:**

Sleep durations shorter or longer than 7 h are associated with cardiovascular diseases. We aimed to investigate the association among sleep duration, risk factors of hypertension, and cardiovascular disease in South Korea using data from a recent large-scale survey.

**Methods:**

Data produced by the Korea National Health and Nutrition Examination Survey (KNHANES) were subjected to multivariate logistic analysis. This cross-sectional, nationally representative survey was conducted from Jan 1 to Dec 31, 2011, by the Korean Center for Disease Control and Prevention. Overall, 6,466 participated. Data were analyzed using STATA version 13.0 (STATA Corp LP).

**Results:**

The participants’ socioeconomic, physical, and lifestyle factors were statistically different between the two age groups (<65 yr and ≥65 yr). Shorter sleep durations were associated with hypertension in individuals younger than 65 yr of age. On the other hand, in participants aged ≥65 yr, both shorter and longer sleep durations were associated with hypertension, while shorter sleep durations were associated with cardiovascular diseases.

**Conclusion:**

Unusual sleep durations are associated with an increased prevalence of cardiovascular disease among Korean adults. The effect of sleep duration appears to be more significant in individuals with hypertension, suggesting that the management of hypertension should be prioritized in patients older than 65 year.

## Introduction

Sleep duration has decreased in the general population over recent years. According to the ‘Sleep in America poll’ conducted by the National Sleep Foundation in 2015, Americans sleep on average approximately 6.9 h on work days, although they indicated that they would prefer to sleep 7.3 h per night on average (1). In Korea, the average amount of sleep was also reported to be 6.8 h in 2014 (2). Unusual sleep durations in modern society can cause various health problems including cardiovascular diseases (CVDs), such as hypertension (HTN), myocardial infarction, angina, and stroke (3,4). Thus, sleep duration is viewed as a major public health concern. Furthermore, sleep duration independently predicted mortality rates (5,6).

According to the statistics on the causes of death in America, non-communicable diseases were responsible for two-thirds of all deaths globally in 2011, and for 60% of all deaths in 2000. The four main non-communicable diseases were CVDs, cancer, diabetes, and chronic lung disease. Furthermore, individuals that died of CVDs, which included 7 million who succumbed to ischemic heart disease and 6.2 million who died of stroke, accounted for a quarter of all deaths (7). In Korea, CVD is the second most cause of death due to non-communicable diseases, after malignancies. Mortality due to CVDs increases significantly at 55 and 65 yr in Korean men and women, respectively (2).

Approximately 26.4% of the world’s adult population suffers from HTN, which accounts for 13.5% of all deaths. The number of people affected has been predicted to grow to more than 1.5 billion people by 2025. Indeed, high blood pressure has been shown to be a powerful determinant of the risks of stroke and ischemic heart disease (8–10).

Unusual sleep durations were positively associated with the incidence of CVDs (11–16). A relationship was reported between self-reported sleep duration and prevalence of HTN (11). In a longitudinal analysis of US National Health and Nutrition Examination survey [NHNES] data from 2006, an association was reported between self-reported short sleep duration and incident HTN (12). A significant association between short sleep duration and prevalence and incidence of HTN was reported and was used to obtain objective measures of sleep duration (13–15). In addition, a significant association was reported between sleep duration and the presence of CVDs in a US national health interview survey (16). Daily sleep duration of ≤7 h was associated with an increased prevalence of HTN (17–19). Moreover, middle-aged participants with sleep duration of ≤ 5 h were at a high risk of developing HTN (17). However, in a community-based prospective study, sleep durations shorter or longer than 6 h were associated with the prevalence of HTN (11). In postmenopausal women, unusual sleep durations were demonstrated to be a risk factor of ischemic stroke (19). Furthermore, sleep deprivation increased the prevalence of myocardial infarction in middle-aged women (20). In the clinical context, many patients with CVD developed HTN (2). However, the association between sleep duration and CVD with HTN remains unclear in Korea. We hypothesized that the relationship between inadequate sleep and the incidence of CVDs is stronger in patients with HTN than in those without, because of the associations found between sleep duration and HTN (21).

The main objective of present study was to determine the nature of the relationships among sleep duration, the risk factors of HTN, and the presence of CVDs using data produced by the Fifth Korean National Health and Nutrition Examination Survey (KNHANES-V; 2011). To achieve this, we first classified the patients according to the socioeconomic, physical, and psychological factors and their lifestyles. Subsequently, we performed comparative analyses on healthy participants (normal controls), participants with HTN, and participants with both HTN and CVD. We also calculated the risk ratios for different sleep durations in the normal, HTN, and HTN with CVD groups.

## Methods

### Study design

This retrospective study was based on a secondary data analysis obtained from the 2011 Korea National Health and Nutrition Examination Survey (KNHANES V, 2011-02CON-06-C) and nationally representative survey performed in Korea.

### Participants

The KNHANES V study involved a health interview, health behavior and nutrition survey, and a medical examination. The 8,518 individuals that participated were enrolled using the stratified sampling method. Of these participants, 6,466 were included in the present study, while the remaining 2,052 were excluded because they had more than two CVDs. We divided the participants into three groups: Normal, HTN, and HTN with CVD. The Normal group included participants who did not have any diagnosis. The HTN group included participants diagnosed by physicians with HTN, while the HTN with CVD group included participants diagnosed with both HTN and CVD (such as angina, myocardial infarction, and stroke).

### Ethics approval

The KNHANES V study was approved by the Institutional Review Board of institution. All participants completed and signed an informed consent form (2011-02CON-06-C).

### Variables

#### Sleep duration

The questionnaire on sleep duration presented to the 6,466 study participants contained the question “On average, how many hours do you sleep per day?” and five possible answers, namely ≤5, 6, 7, 8, or ≥9 h. In the present study, ‘normal’ sleep was defined as 7 h per day, which is in line with a previous study (22). Sleep duration of shorter and longer duration on a basis of 7 h were considered ‘unusual’ sleep durations.

#### Socioeconomic factors

The socioeconomic factors were also recorded. The questionnaire contained items on sex, age (<65 or ≥65 yr), education (high school or lower, high school or equivalent, college graduate or higher), employment (unemployed or employed), marital status (unmarried or married), and monthly income (<1,000, 1,000–2,000, 2,000–3,000, or ≥3,000 US dollars; 1 dollar=1,000 Korean Won).

#### Physical factors

Physical factors, such as body mass index (BMI), presence of diabetes, glycosylated hemoglobin 1Ac (HbA1c), hyperlipidemia, and triglyceride levels were recorded. The BMI, HbA1c, and triglyceride levels were analyzed by anthropometric measurement and blood tests. The presences of diabetes and hyperlipidemia were determined based on the physician’s diagnosis.

#### Psychological factors

The psychological factors investigated included stress and depression. The questionnaire on stress contained the question “How much stress do you experience in daily life?” and the stress levels were divided into five categories. The presence of depression was determined based on the physician’s diagnosis. The quality of life was measured using the EQ-5D instrument.

#### Lifestyle factors

Lifestyle factors included smoking, drinking, exercise, and eating habits. The lifestyle factors were self-reported. The smoking and drinking statuses were categorized according to the exposure experience such as “non,” “ex,” and “current.” Exercise frequencies were quantified as numbers of exercise days per week.

#### Data analysis

The data analysis was conducted using STATA version 13.0 (STATA Corp LP). Categorical variables were expressed as numbers and percentages, and continuous variables were expressed as means ± standard deviations. Differences between groups were evaluated using the χ^2^ test. Multivariable-adjusted logistic regression analysis was performed to determine the odds ratios (ORs) and 95% confidence intervals (CIs) of categories of sleep duration. Multivariate analysis was adjusted for sex, obesity, diabetes, and hyperlipidemia. Factors included in the analysis were chosen by reviewing related literature. According to previous sleep duration related studies, age, sex, depression, diabetes, and obesity have been suggested to be confounding factors. Statistical significance was accepted for *P*-values <0.05.

## Results

### General characteristics

The baseline characteristics of the 6,466 study participants are summarized in Tables 1 and 2. Overall, 4916 participants were younger than 65 yr of age. Of these, 557 participants were diagnosed with HTN and 46 were diagnosed with both HTN and CVD. The socioeconomic, physical, and lifestyle factors were statistically different among the groups (Table 1).

**Table 1:** General characteristics of 6466 individuals participated in this study

***Category***			***Normal (n=5,056)***	***HTN (n=1,218)***	***HTN with CVD (n=192)***	***χ^2^ or F***	***P-value***
Socio-economic	Gender	Men	2,178 (78.3)	508 (18.3)	95 (3.4)	4.13	0.127
		Women	2,878 (78.1)	710 (19.3)	97 (2.6)		
	Age(yr)	<65	4,313 (87.7)	557 (11.3)	46 (0.9)	1.1e+03 (1100)	<0.001 ^***^
		≥65	743 (47.9)	661 (42.7)	146 (9.4)		
	Education	High school or lower	1,247 (57.2)	810 (37.1)	125 (5.7)	714.19	<0.001^***^
		High school	1,666 (84.4)	268 (13.6)	40 (2.0)		
		College or higher	1,601 (90.7)	139 (7.9)	26 (1.5)		
	Employment	Unemployed	2,829 (81.3)	594 (17.1)	57 (1.6)	143.94	<0.001^***^
		Employed	1,686 (69.0)	623 (25.5)	134 (5.5)				
	Marrital Status	Unmarried	4,163 (75.0)	1,197 (21.6)	190 (3.4)	226.46	<0.001^***^
		Married	871 (97.4)	21 (2.4)	2 (0.2)		
	Income	<100	1,170 (77.3)	296 (19.6)	41 (3.1)	6.49	0.371
		100∼200	1,258 (76.9)	320 (19.6)	57 (3.5)		
		200∼300	1,295 (78.7)	301 (18.3)	49 (3.0)		
		≥300	1,269 (79.7)	287 (18.0)	37 (2.3)		
physical	BMI	Mean (SD)	23.2 (3.3)	25.0 (3.4)	25.0 (3.1)	148.30	<0.001^***^
		Diagnosis of diabetes	187 (36.9)	256 (50.5)	64 (12.6)	585.4	<0.001^***^
	HbA1c	Mean (SD)	5.6 (0.8)	6.1 (0.9)	6.3 (1.0)	196.54	<0.001^***^
		Diagnosis of hyperlipidemia	245 (36.8)	342 (51.4)	78 (11.7)	771.63	<0.001^***^
	TG	Mean (SD)	126.6 (105.3)	153.8 (107.9)	152.1 (99.3)	33.07	<0.001^***^
Psychological	Stress	A great deal	534 (67.0)	274 (28.3)	45 (4.7)	210.89	<0.001^***^
		Much	2,640 (78.0)	647 (19.1)	98 (2.9)		
		Somewhat	1,035 (79.1)	239 (18.3)	34 (2.6)		
		Little	199 (75.1)	52 (19.6)	14 (5.3)		
		Never	534 (98.7)	6 (1.1)	1 (0.2)		
	Depression	Yes	579 (72.3)	178 (22.2)	44 (5.5)	29.26	<0.001^***^
	QoL (EQ-5D)	Mean (SD)	0.95 (0.1)	0.89 (0.2)	0.81 (0.2)	220.81	<0.001^***^
Lifestyle	Smoking	Nonsmoker	2,682 (77.1)	703 (20.2)	95 (2.7)	81.29	<0.001^***^
		Ex-smoker	826 (68.1)	317 (26.1)	70 (5.8)		
		Current smoker	1,012 (82.2)	193 (15.7)	26 (2.1)		
	Drinking	Nondrinker	484 (59.7)	277 (34.2)	50 (6.2)	212.40	<0.001^***^
		Ex-drinker	568 (68.3)	217 (26.1)	47 (5.7)		
		Current drinker	3,452 (80.9)	718 (16.8)	95 (2.2)		
	Exercise	Non	2,794 (73.4)	874 (23.0)	1387 (2.6)	54.72	<0.001^***^
		1∼2	821 (83.9)	137 (14.0)	21 (2.2)		
			≥3	904 (79.4)	202 (17.8)	32 (2.8)		
	Eating habits (skip)	Non	560 (83.3)	104 (15.5)	8 (1.2)	15.02	0.001^***^
		Skip ≥1	4,496 (77.6)	1,114 (19.1)	184 (3.2)		

* *P*<0.05 ;

** *P*<0.01,

*** *P*<0.001; HTN, hypertension; HTN with CVD, hypertension with cardiovascular diseases; BMI, body mass index; HbA1c, glycosylated hemoglobin; TG, triglyceride; QoL, quality of life

On the other hand, 1550 participants were older than 65 yr of age. Of these, 661 were diagnosed with HTN, while 146 were diagnosed with both HTN and CVD. The socioeconomic, physical, and lifestyle factors were statistically different among the groups (Table 2).

### Sleep duration and CVDs

Tables 3 and 4 summarize the ORs and 95% CIs for the prevalence of HTN and HTN with CVD for the five categories of sleep duration. In the present study, sex, obesity, diabetes, and hyperlipidemia differed significantly between the two age groups (<65 yr and ≥65 yr) and among the Normal, HTN, and HTN with CVD groups. Table 3 summarizes the ORs and 95% CIs for the prevalence of HTN and HTN with CVD in the younger group. In this group, the unadjusted OR for the prevalence of HTN in those with sleep duration ≤5 h was 1.76, relative to those with sleep duration of 7 h. After adjusting for sex and physical factors, a significant association remained between the prevalence of HTN and sleep duration ≤5 h (OR=1.57; 95% CI=1.16–2.14), but not with sleep duration >5 h. In contrast, no significant association was found between sleep duration and the prevalence of HTN with CVD.

**Table 2:** Characteristics of subjects across categories of sleep duration

***Variable***	***≤5***	***6***	***7***	***8***	***9≤***
<65(yr)					
Normal	446(11.41)	1,064(27.23)	1,219(31.19)	901(23.06)	278(7.11)
HTN	85(15.29)	147 (26.44)	175(31.47)	118(21.22)	31(5.58)
HTN with CVD	8(17.39)	14(30.43)	13(28.26)	7(15.22)	4(8.70)
≥65(yr)					
Normal	174(28.34)	142(23.13)	124(20.20)	114(18.57)	60(9.77)
HTN	199(30.43)	170(25.99)	129(19.72)	97(14.83)	59(9.02)
HTN with CVD	45(31.03)	33(22.76)	32(22.07)	23(15.86)	12(8.28)

**Table 3:** Odds ratios for CVD in relation to reported sleep duration (<65 yr)

***Category***		***N***	***OR***	***95%***		***P-value***
Unadjusted						
Only HTN	≤5	85	1.76	1.33	2.33	0.002
	6	147	1.27	1.01	1.61	0.449
	7	175		Reference		
	8	118	1.21	0.94	1.55	0.409
	9≤	31	1.03	0.09	0.13	0.680
HTN with CVD	≤5	8	2.24	0.92	5.44	0.121
	6	14	1.64	0.77	3.51	0.484
	7	13		Reference		
	8	7	0.97	0.39	2.44	0.834
	9≤	4	1.80	0.58	5.55	0.562
Adjusted ^†^						
Only HTN	≤5	85	1.61	1.19	2.17	0.002
	6	147	1.10	0.86	1.42	0.449
	7	175	Reference				
	8	118	1.12	0.86	1.46	0.409
	9≤	31	0.91	0.59	1.41	0.680
HTN with CVD	≤5	8	2.06	0.83	5.16	0.121
	6	14	1.32	0.60	2.90	0.484
	7	13	Reference				
	8	7	0.90	0.35	2.32	0.834
	9≤	4	1.42	0.43	4.64	0.562

† Adjusted = adjusted physical factors, such as presence of diabetes and presence of hyperlipidemia

Table 4 summarizes the ORs and 95% CIs in relation to the prevalence of HTN and HTN with CVD in the older group (≥ 65 yr). In this group, the unadjusted OR for the prevalence of HTN in those with sleep durations shorter and longer than 7 h were calculated with respect to those with sleep duration of 7 h. The unadjusted ORs of those with sleep duration ≤ 5 and 6 h were almost twice those of participants who slept for 7 h. After adjusting for sex and physical factors, significant associations remained between the prevalence of HTN and shorter or longer sleep duration (≤5 h, OR=1.85; 6 h, OR=2.01; 8 h, OR=1.60; ≥9 h, OR=1.62). In contrast, significant associations remained between the prevalence of HTN with CVD and shorter sleep duration (OR=1.68; 95% CI=2.81).

**Table 4:** Odds ratios for CVD in relation to reported sleep duration (≥65)

***Category***		***N***	***OR***	***95%***		***P-value***
Unadjusted						
Only HTN	≤5	199	1.78	1.59	2.85	<0.001
	6	170	1.27	1.64	3.02	<0.001
	7	129	Reference			
	8	97	1.21	1.13	2.23	0.008
	9≤	59	1.03	1.21	2.77	0.004
HTN with CVD	≤5	45	2.24	1.22	3.23	0.006
	6	33	1.64	1.05	3.01	0.031
	7	32	Reference			
	8	23	0.97	0.87	2.75	0.138
	9≤	12	1.80	0.75	3.15	0.243
Adjusted ^†^						
Only HTN	≤5	199	1.90	1.41	2.57	<0.001
	6	170	2.05	1.50	2.81	<0.001
	7	129	Reference			
	8	97	1.62	1.14	2.30	0.007
	9≤	59	1.70	1.11	2.62	0.016
HTN with CVD	≤5	45	1.66	1.00	2.76	0.050
	6	33	1.55	0.90	2.67	0.115
	7	32	Reference			
	8	23	1.65	0.91	3.00	0.098
	9≤	12	1.40	0.66	2.95	0.377

† Adjusted = adjusted physical factors, such as presence of diabetes and presence of hyperlipidemia

## Discussion

Approximately 80 million Americans suffer from HTN (21), which is a major risk factor of myocardial infarction, heart attack, stroke, and congestive heart failure (22). Furthermore, HTN is associated with lifestyle factors, such as economic state, mental stress, eating habits, and sleep (23). The relationship between sleep and HTN has been previously demonstrated. Short sleep duration was associated with the prevalence of HTN among middle-aged (32 to 59 yr) Americans (12). Another cross-sectional analysis performed in Britain reported a significant association between short sleep duration and HTN (25). In the Sleep Heart Health Study, short sleep durations were associated with HTN prevalence (11). Our present findings corroborated those of previous studies. Our results indicated that short sleep duration was positively associated with HTN in participants younger than 65 yr of age. In addition, a positive association was observed between short sleep duration and HTN with CVD in participants aged ≥65 yr in the 2011 KNHANES study. This observation is in striking contrast with other studies. Indeed, conflicting results between sleep duration and hypertension have been reported in the elderly.

Sleep and high blood pressure do not correlate in the elderly population (25–28). This is inferred from the fact that the range of elderly group is different and that the self-report questionnaire would have reduced the accuracy of the data collection. Unusual sleep durations certainly act as risk factors for high blood pressure. To prove this, studies have investigated the relationship between sleep duration and HTN and reported that the efficacy of sleep duration and sleep improvement reduced blood pressure. These effects were influenced affected by sex, location of the population, and operational definition of sleep duration (28).

The most remarkable aspect of the present study was our finding of a positive association between short sleep duration and hypertension in participants aged ≥ 65 yr, which is not consistent with previous findings (27). Sleep duration and HTN were not associated in Koreans aged ≥65 yr. However, an association between short sleep duration and the prevalence of HTN in Koreans aged <65 yr have been reported after adjusting for sex, obesity, smoking status, alcohol consumption, physical activity, depressive symptoms, diabetes mellitus, and stroke (OR=1.314; 95% CI=1.01–1.71) (27).

Short sleep durations were associated with a higher risk for HTN and with longitudinal effects to HTN (21, 29). However, these previous studies did not adjust the results for age or comorbidity diseases such as CVD, and the referred sleep hour was not consistent.

In contrast to previous studies, we subdivided outpatients with HTN into two groups based on the presence or absence of CVD. We believe this subdivision is meaningful and adds value to our results because HTN is a risk factor of CVD (9,10). Nevertheless, our results also suggest the need for further research on specific aspects of CVD. Furthermore, other studies conducted on middle-aged women have concluded that unusual sleep durations were a risk factor of ischemic stroke and myocardial infarction (30,31).

In the present study, we adjusted for sex, obesity, diabetes, and hyperlipidemia based on the available literature indicating that HTN and CVD are related. In a previous review study about sleep duration and health, the analysis was adjusted for age, sex, obesity, depression, and lifestyle (smoking, alcohol consumption, and exercise) (32). Sex and obesity were adjusted and we noted a significant association between short sleep duration and hypertension (29). The effect of sleep duration may be more significant in individuals with HTN, suggesting that the management of HTN should be prioritized in patients older than 65 yr.

## Limitations

First, some of the cardiovascular risk factors could not be considered because the original data were previously collected by the Korea Centers for Disease Control and Prevention. Second, although many previous studies have reported that self-reported data are not prone to major reporting errors (23–25), the self-reported sleep duration data may have included errors. Third, the body weights were not considered, and we cannot be certain of how representative these data are. Fourth, the small sub-sample size may be associated with an increasing risk of Type I error.

## Conclusion

The short sleep duration is associated with an increased prevalence of HTN among Korean adults. In the case of HTN with CVD, there was an association between sleep duration and the disease prevalence in the older group. Further research is needed using a longitudinal follow-up design in order to establish whether there is a causal relationship between sleep duration and the prevalence of CVD.

## Ethical considerations

Ethical issues (Including plagiarism, informed consent, misconduct, data fabrication and/or falsification, double publication and/or submission, redundancy, etc.) have been completely observed by the authors.
